# The Thoracic Absorption Dose and Secondary Tumor Risk Caused by Different Imaging Methods in Image‐Guided Particle Radiotherapy

**DOI:** 10.1111/1759-7714.70102

**Published:** 2025-06-16

**Authors:** Qinghao Cui, Xianrui Yan, Chengqiang Li, Jian Zhu, Jun Ma, Tingting Liu

**Affiliations:** ^1^ Linyi Hospital of Traditional Chinese Medicine Department of Radiation Oncology Physics & Technology Linyi China; ^2^ Department of Engineering Physics Tsinghua University Beijing China; ^3^ Shandong Provincial Key Medical and Health Laboratory of Pediatric Cancer Precision Radiotherapy Shandong Cancer Hospital Jinan China; ^4^ Laboratory of Image Science and Technology, Jiangsu Provincial Joint International Research Laboratory of Medical Information Processing, Centre de Recherche en Information Biomédicale Sino‐français (CRIBs) Southeast University Nanjing People's Republic of China; ^5^ Department of Radiation Oncology Physics & Technology Shandong Cancer Hospital and Institute, Shandong First Medical University and Shandong Academy of Medical Sciences Jinan China; ^6^ Cancer Hospital of Shandong First Medical University Jinan China; ^7^ Sun Yat‐Sen University Cancer Center Gansu Hospital Lanzhou China

**Keywords:** CBCT, LAR, OSLD, particle radiotherapy, thoracic dose

## Abstract

**Background:**

Image‐guided radiation therapy uses imaging methods such as CBCT to effectively improve treatment precision. Kilovoltage‐imaging technology provides high soft tissue contrast at low doses, whereas megavoltage‐imaging technology better displays deep and bony structures at high doses. Proton therapy is more sensitive to tissue density and positional accuracy, so it requires more stringent image guidance and higher precision than traditional X‐ray therapy.

**Objective:**

This study evaluates radiation doses from CBCT systems (TrueBeam, Halcyon, ProBeam, TOMO) in both adult and pediatric phantoms, measuring dose variations and predicting secondary tumor risks using a radiobiological model.

**Methods:**

Absorbed doses in organs of adult and pediatric phantoms were measured with OSLDs across imaging systems. The risk of secondary tumors was estimated using the BEIR VII model.

**Results:**

Halcyon 2.0 and TOMO's MV‐level imaging systems showed significantly higher doses than KV‐level systems. Pediatric patients received 2–3 times higher doses than adults. In KV‐level imaging, Halcyon 2.0 resulted in the highest lung tissue dose in both age groups (17.464 mGy for pediatric, 9.109 mGy for adult), whereas ProBeam had the lowest (6.844 and 4.073 mGy, respectively). The lifetime attributable risk for lung cancer correlated with the dose, with higher risks in children.

**Conclusions:**

Higher radiation doses lead to greater secondary tumor risk, with the risk being more pronounced in pediatric patients. Continuous thoracic CBCT can deliver up to 1 Gy in thoracic organs, posing a significant risk of secondary tumors, especially in younger patients. Careful consideration of this risk is essential in treatment planning.

## Graphical abstract

This study compared the organ doses and lifetime attributable risk (LAR) of secondary cancers of four commonly used CBCT systems (TrueBeam, Halcyon, ProBeam, and TomoTherapy) in adult and pediatric phantoms. The results showed that MV‐level imaging was higher than KV‐level imaging, and the doses of Halcyon and TomoTherapy systems were higher, whereas the ProBeam system was the lowest; the organ doses in the pediatric phantom were significantly higher than those in adults, and the risk of LAR was also greater. Halcyon had the highest LAR in the thyroid and lungs in children, whereas ProBeam had the lowest LAR in adults. This study emphasizes that there are significant differences in radiation dose and secondary tumor risk among different image‐guided systems, suggesting that image dose optimization should be emphasized in clinical radiotherapy, especially in pediatric patients. The results support the balance between image quality and radiation risk when selecting imaging systems and scanning schemes, giving priority to low‐dose schemes and promoting the development of safer image‐guided technologies. For young patients with good prognosis, clinical decisions should seek a reasonable balance between precise positioning and potential long‐term risks.

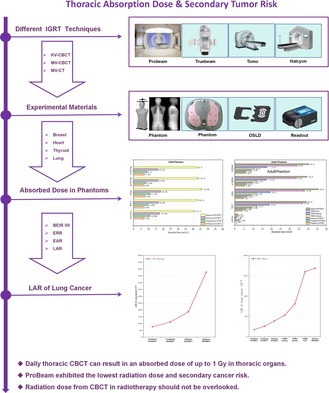

## 1 Introduction

IGRT is essential for improving the precision of radiotherapy, enabling correction of setup errors, real‐time dose monitoring, and adaptive planning. Among available techniques, CBCT is the most widely used and is integrated into most modern linear accelerators [1].

Radiotherapy imaging can be categorized into kV and MV modalities based on beam energy. kV imaging provides superior soft tissue contrast with a relatively low radiation dose, whereas MV imaging offers better penetration and bone visualization but lower soft tissue contrast and higher exposure.

Compared with X‐ray radiotherapy, proton radiotherapy requires more rigorous image guidance and daily verification to ensure precise dose delivery due to its sensitivity to tissue heterogeneity and positioning accuracy. This is particularly important in pediatric patients [2], who often require frequent image guidance due to limited compliance and longer life expectancy, increasing the emphasis on minimizing cumulative dose and long‐term risk.

Most patients undergo multiple imaging sessions during therapy, especially for tumors affected by respiratory motion, which increases the risk of tumor misalignment. These scans contribute to additional radiation exposure [3], often overlooked in dosimetry assessments. This study investigates the absorbed dose and secondary tumor risk associated with different imaging systems. Using optically stimulated luminescence dosimetry and a radiobiological model, the research aims to support decisions to enhance patient quality of life.

## 2 Materials and Methods

### 2.1 Experimental Equipment

#### 2.1.1 Imaging‐Guided Devices

The TrueBeam system (Varian) includes a linear accelerator, respiratory gating, and a kV‐CBCT system (OBI) with an X‐ray source and flat‐panel detector on retractable arms. Halcyon 2.0 (Varian) is a 6 MV linear accelerator with daily CBCT for precise image‐guided radiotherapy. TomoTherapy uses a single MV‐level radiation source (3.5 MV for imaging, 6 MV for treatment) with an image plate aligned to the treatment center for efficient guidance.

Proton therapy, led by Varian's ProBeam in this study, has shown positive outcomes in pediatric cancers [4]. Studies indicate that the ProBeam system offers superior heart protection [5] in pediatric whole‐lung proton therapy. It features two kilovoltage CBCT systems [6] with retractable detection plates and X‐ray sources mounted on the rotating frame.

#### 2.1.2 Optically Stimulated Luminescence Dosimeter (OSLD) System

The OSLD system (RadPro, Germany) includes myOSLchips, a portable reader, an annealing device with 24 blue LEDs, and calibration software. Similar to nanoDot dosimeters, these BeO‐based OSLDs (4.7 × 4.7 mm) are embedded in plastic holders, identified by QR codes, and require postmeasurement annealing.

BeO dosimeters offer high sensitivity, thermal stability, and a broad dose range (0.05 mGy–10 Gy). They allow repeatable readings, resist environmental factors, and have an atomic number (7.2) close to human soft tissue [7].

#### 2.1.3 Anthropomorphic Phantoms

**FIGURE 1:**
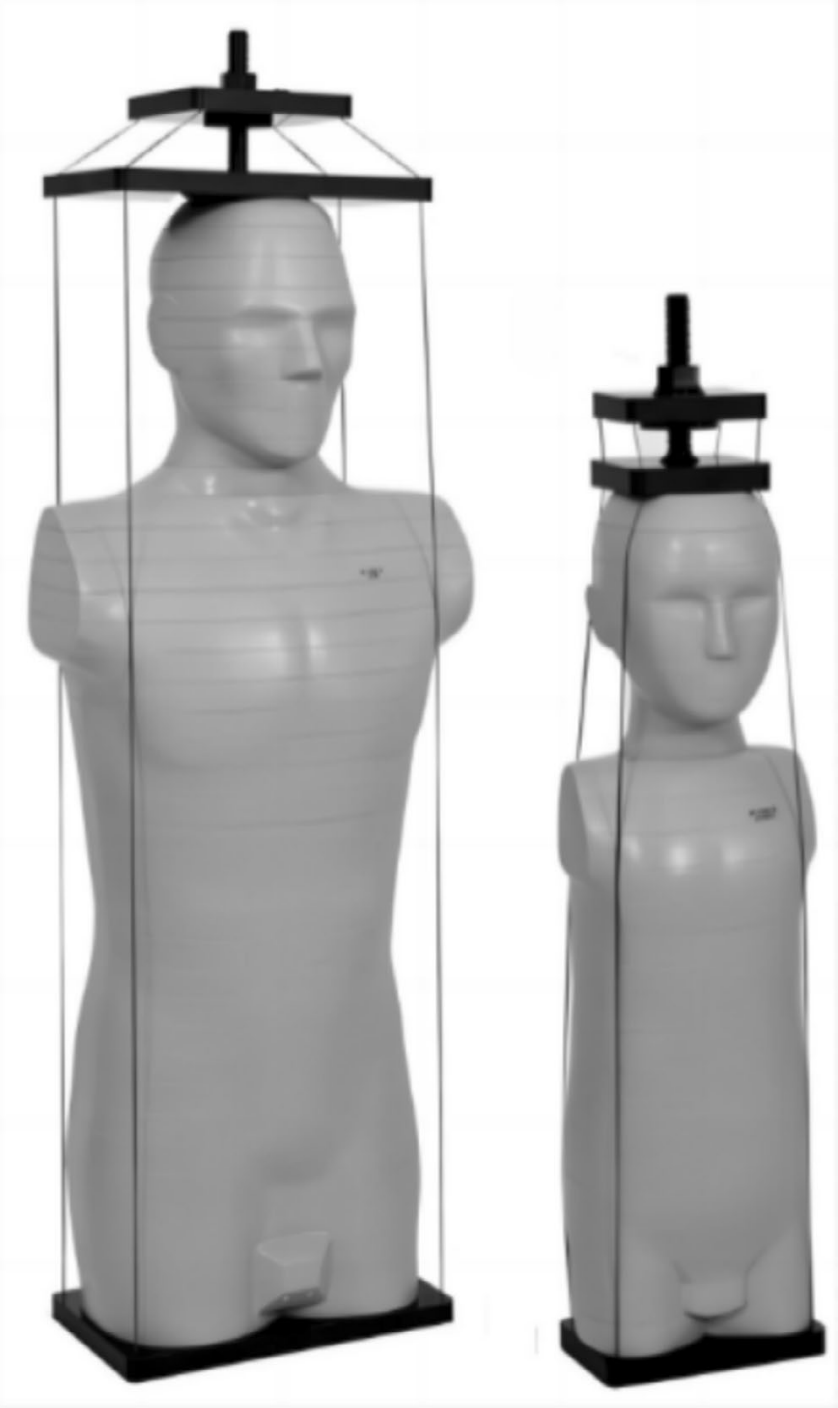
Anthropomorphic phantoms.

The adult model (701‐D) and the 5‐year‐old child model (705‐D) of CIRS ATMO are used in this study, as shown in Figure 1. The phantom size is primarily based on the ICRP 23 and ICRU 48 reports [8]. It is capable of measuring 22 internal organs, each with multiple measurement holes that can accommodate adapters for different types of dosimeters such as TLDs and OSLDs.

### 2.2 Scale Design

**FIGURE 2:**
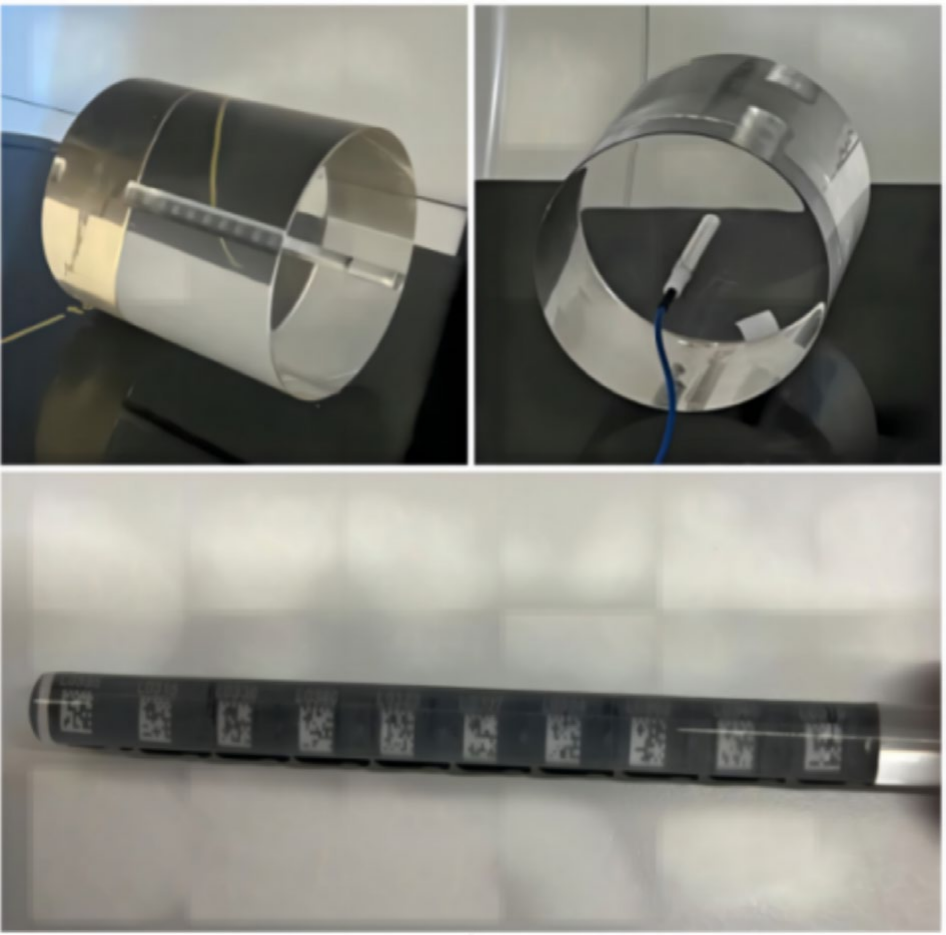
Custom‐made phantom.

This study presents the design of a specialized module for OSLD dose calibration, as illustrated in Figure 2, using Polymethyl Methacrylate (PMMA) as the insert material. The insert consists of two distinct components: The first is a cylindrical rod with a bottom diameter of 10 mm and a length of 150 mm. A 2‐mm‐thick groove runs along its diameter, providing a secure clamp for the OSLD dosimeter. The second component is a custom‐designed insert with a bottom diameter of 160 mm and a height of 150 mm. A central hole, 10 mm in diameter and 125 mm in depth, is precisely aligned along the axis of the cylinder.

This study outlines the absolute dose calibration process for KV‐level imaging equipment using a custom‐made phantom. Two measurement methods, CT ionization chamber and OSLD, were utilized. The phantom was positioned at the center of the treatment bed to reduce radiation attenuation errors. For the CT ionization chamber [9], measurements were taken three times for averaging. For OSLD, 10 OSLDs were placed in a PMMA insert in the phantom. After 15 min, data were collected, the signal erased, and background values reassessed, with the procedure repeated three times for accuracy. According to the TRS469‐2009 report [10], under identical measurement conditions, the conversion factor (CF) formula is used to determine the absolute dose value. This value is measured by the CT ionization chamber and recorded by the OSLD:

(1) $${\mathrm{CF}}_{\mathrm{KV}}=\frac{M∙N_{k}∙N_{c}∙d^{-1}}{M_{\text{mean}}}=\frac{D_{\mathrm{cal}}∙d^{-1}}{M_{\text{mean}}}$$

$M_{\text{mean}}$, mean OSLD readings (mGy); $N_{c}$, calibration factors provided by the metrology institute; $N_{k}$, air‐to‐air specific kinetic energy calibration factor; *d*, CT chamber length (cm); M, CT ionization chamber readings adjusted for temperature and atmospheric pressure (mGy cm).

A medical electron linear accelerator, with a flatness symmetry of less than 2%, is used to calibrate the absolute dose of MV‐level imaging equipment. A 5 cm thick solid water block is placed on the treatment bed, and the OSLD is carefully positioned at the center of the solid water surface. The treatment bed coordinates are adjusted to ensure the OSLD is near the center of the radiation field. Another 5 cm thick solid water block is then placed on top of the OSLD. The SSD is set to 100 cm, and the radiation field size is maintained at 10 × 10 cm, with an output of 100 MU. The OSLD readings are taken 15 min postirradiation, with measurements repeated three times and subsequently averaged.

The solid water block is then adjusted, and the absolute dose at a depth of 5 cm below the surface is measured using a PTW ionization chamber (Farmer, 0.6 cc). These measurements are also repeated three times and averaged.

The imaging beams used in this study had energy levels of 3.5 and 6 MV. Since BeO exhibits a consistent energy response curve within the 1–10 MV range, it can be inferred that the energy response factors for absolute dose calibration across the two distinct imaging devices are approximately equal.

(2) $${\mathrm{CF}}_{\mathrm{MV}}=\frac{D_{\mathrm{cal}}}{M_{\text{mean}}}$$

### 2.3 Measurements

**FIGURE 3:**
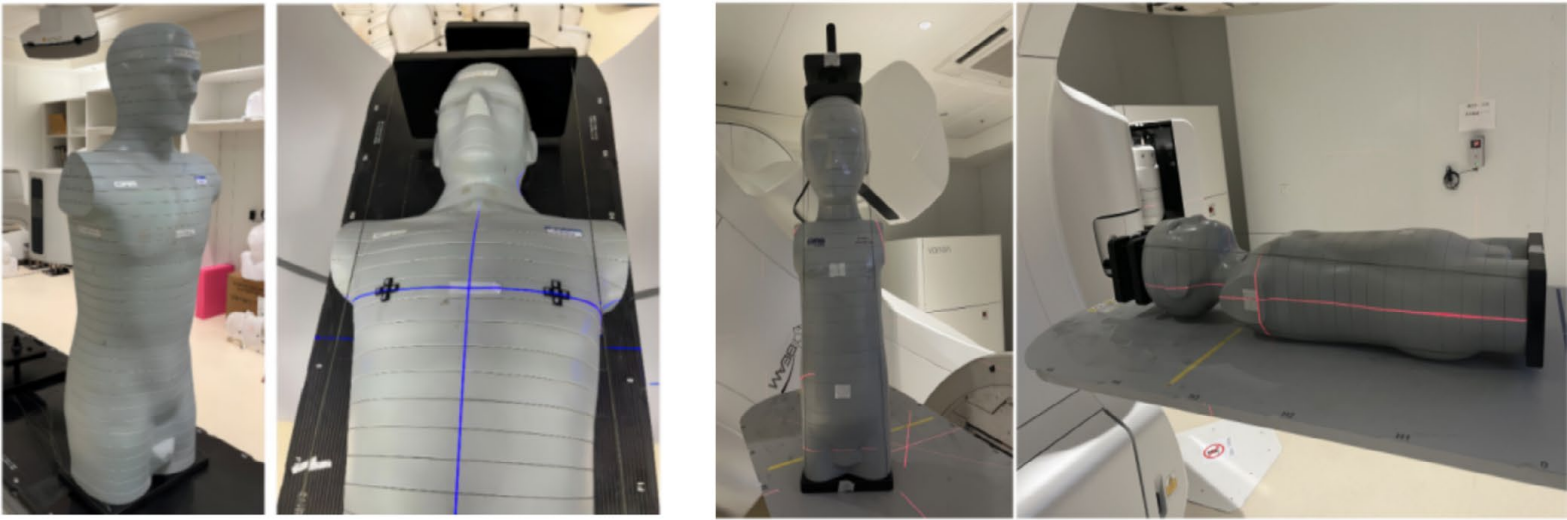
Phantom experimental measurement.

The CIRS simulated human body model should be assembled, with the left and right positions of the treatment bed set to zero. As shown in Figure 3, the simulation model should then be placed flat on the treatment bed, with adjustments made to ensure the laser is aligned with the model's midline. Markings should be made on the head, chest, and abdomen of the model. The height and bed entry values should be adjusted so that the laser aligns with the line connecting the model's two breasts. Additionally, lines should be marked at the intersection of the laser beams on both sides and at the center of the model, as shown in Figure 3.

Each organ contains multiple measurement points, from which an average value is derived to represent the dose for that specific organ. The calculation methodology is as follows:

(3) $$D_{\text{organ}}=\frac{1}{n}\left(\sum_{i}{\bar{D}}_{i}\right)$$

$D_{\text{organ}}$ represents the absorbed dose of the organ measured using optically stimulated luminescence; ${\bar{D}}_{i}$ is the average value (mGy) of the absorbed dose at that point measured by the optically stimulated luminescence dosimeter i in the phantom; n is the number of measurement points within a specific organ. The absorbed dose at each measurement point is calculated using the following Formula (4):

(4) $$D_{i}=D_{x}∙{\mathrm{CF}}_{\mathrm{kv}}∙{\mathrm{TF}}_{\mathrm{en}}$$

$D_{x}$ represents the reading of the measured OSLD; ${\mathrm{CF}}_{\mathrm{kv}}$ is the kilovolt‐level radiation absolute dose calibration factor, which ${\mathrm{TF}}_{\mathrm{en}}$ is the ratio of the tissue organ to air mass energy absorption coefficient, calculated as:

(5) $${\mathrm{TF}}_{\mathrm{en}}={\left(\frac{\mu_{\mathrm{en}}}{\rho }\right)}_{\text{organ}}/{\left(\frac{\mu_{\mathrm{en}}}{\rho }\right)}_{\mathrm{air}}$$

Radiation doses from KV‐CBCT (TrueBeam, Halcyon 2.0, ProBeam) were measured using an adult simulation phantom. The phantom was positioned supine, aligned centrally on the treatment bed, with the laser aligned to its marking line. The standard clinical chest scanning mode was used, and each test was repeated three times. The Halcyon 2.0 uses arc rotation for 15‐s MV‐CBCT imaging in low (5 MU) dose modes.

In the adult model experiment, TomoTherapy served as a control and featured three scanning modes: fine, normal, and coarse. The average absorbed dose at all measurement points within the organ was calculated as the organ's absorbed dose using the formula (3). n represents the number of measurement points for the organ; ${\bar{D}}_{i}$ is the average absorbed dose at point i. The specific calculation method for the absorbed dose at each measurement point was calculated using the following formula:

(6) $$D_{i}=D_{x}∙{\mathrm{CF}}_{\mathrm{MV}}∙{\mathrm{TF}}_{\mathrm{en}}$$

$D_{x}$ represents the reading of the OSLD; ${\mathrm{CF}}_{\mathrm{MV}}$ is the megavoltage absolute dose calibration factor, as shown in Formula (2); ${\mathrm{TF}}_{\mathrm{en}}$ is the ratio of the tissue organ to air mass energy absorption coefficient, given by the formula (5).

The KV and MV imaging modes of TrueBeam, Halcyon, and ProBeam were measured using a 5‐year‐old child phantom. The experimental parameters, procedures, and data collection methods were consistent with those used in the adult phantom experiments.

### 2.4 BEIR VII Model

The BEIR VII model, a report published by the U.S. National Council on Radiation Protection (NCRP) [11], examines the health effects of radiation, with a particular focus on the impact of low‐dose radiation on human health [11]. This model is based on the assumptions of the linear no‐threshold (LNT) model [12] and uses data derived from the study of Japanese atomic bomb survivors [13]. It incorporates factors such as gender, age, and genetics to evaluate the carcinogenic risk and noncancer effects of lowdose radiation exposure in humans.

According to the BEIR VII model, the ICRP 60 [14] stipulates that risk assessments for all solid cancers, excluding thyroid and breast cancer, should be computed using a weighted summation of both the absolute risk model and the relative risk model. Conversely, breast cancer should be assessed using the absolute risk model, whereas thyroid cancer should be evaluated using the relative risk model. Notably, for lung cancer, the excess absolute risk (${\mathrm{LAR}}_{\mathrm{EAR}}$) carries a weighting of 70%, whereas the excess relative risk (${\mathrm{LAR}}_{\mathrm{ERR}}$) is assigned a weighting of 30% [11]. The evaluation of the parameters for the lung cancer incidence model is presented in Table 1:

**TABLE 1:** Evaluation of parameters for lung cancer incidence models [11].

Organ	Lung			
	$\beta_{M}\left(95\%\mathrm{CI}\right)$	$\beta_{F}\left(95\%\mathrm{CI}\right)$	γ	η
ERR	0.32 (0.15, 0.70)	1.4 (0.94, 2.1)	−0.30	−1.4
EAR	2.3 (1.1, 5.0)	3.4 (2.3, 4.9)	−0.41	5.2 (3.8, 6, 6)

To compute the lifetime cancer risk (LAR) using expected absolute excess risk (EAR) and expected relative excess risk (ERR), it is essential to obtain age‐specific mortality data from the Seventh National Population Census of China [11]. The Farlee Death Mortality Probability formula should then be applied to determine death probabilities across various ages and genders, followed by the derivation of the Chinese population life table [15]. This study will use survival rates from the life table for different ages and genders.

Using cancer statistics from the Chinese population, we can determine the lung cancer incidence rate across different age groups and genders within China. By integrating the Chinese population life table [19], lung cancer incidence data from the National Cancer Center, the BEIR VII model, and the measured dose data, we can calculate the carcinogenic risk for the Chinese population using the following formula:

(7) $$\mathrm{LAR}=M\left(D,e,a\right)∙S\left(a\right)/S\left(e\right)$$

In the formula: D represents the absorbed dose of the measured organ; e is the age at exposure; a is the age of arrival; $S\left(a\right)$ is the survival probability up to age a, and $S\left(a\right)/S\left(e\right)$ is the probability of surviving from age e to age a after radiation exposure. $M\left(D,e,a\right)$ represents the ${\mathrm{LAR}}_{\mathrm{EAR}}$ obtained at age a from exposure to dose D at age e.

(8) $$M\left(D,e,a\right)=\mathrm{ERR}\left(D,e,a\right)∙I_{I}\left(a\right)$$

In the formula: $M\left(D,e,a\right)$ is the excess absolute risk, $\mathrm{ERR}\left(D,e,a\right)$ is the excess relative risk for different doses D, different ages at exposure, and different ages at arrival, and $I_{I}\left(a\right)$ is the baseline incidence of lung cancer in the whole population at the age of arrival.

In Formula (9), the weighting factors for specific tumors are recommended based on the BEIR VII model. For lung cancer, an excess absolute risk is assigned a weight of 0.7, whereas an excess relative risk is assigned a weight of 0.3. The dose rate correction factor DDREF is set to 1.5:

(9) LAREAR=∑aD∙βsexpγe*a60η∙Sa/Se

(10) LARERR=∑aD∙βsexpγe*a60η∙Sa/Se∙IIa

After weighting, the LAR of the tumor is:

(11) $$\mathrm{LAR}={\left({\mathrm{LAR}}_{\mathrm{EAR}}\right)}^{0.7}\times {\left({\mathrm{LAR}}_{\mathrm{ERR}}\right)}^{0.3}/\text{DDREF}$$

$e^{*}$, related to age of exposure; *e* ≥ 30: e* = 0, *e* < 30: e* = (*e*−30)/10; $\beta_{s}$, related to gender; γ, perdecade increase in age at exposure over the range 0–30 years (γ); η, reaching the age quantification index.

## 3 Results

This study focused on four organs of interest: the thyroid, lungs, breasts, and heart. Each organ was assessed at multiple measurement points, with the absorbed dose of the organ tissue calculated as the mean value across all measurement points within that specific organ.

### 3.1 Measurement of Adult Phantom

**FIGURE 4:**
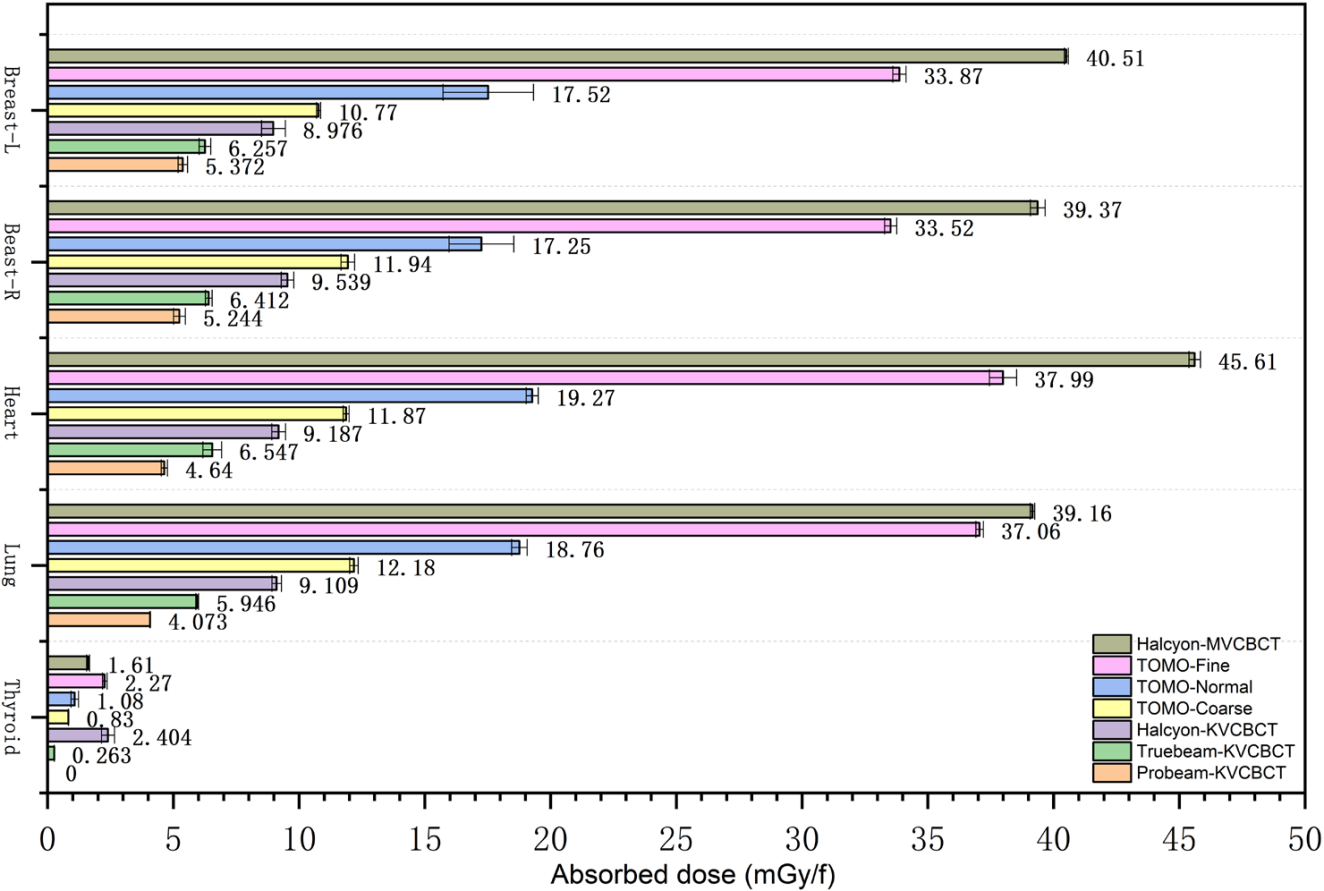
Absorbed dose of adult phantom induced by different imaging devices in IGRT.

Phantom measurements show that MV imaging delivers higher absorbed doses than KV imaging. Among KV devices, Halcyon had the highest lung dose (9.1 mGy), followed by TrueBeam (5.946 mGy) and ProBeam (4.073 mGy). For MV imaging, Halcyon‐MV‐CBCT had the highest dose, whereas TOMO‐Coarse mode had the lowest (3.916 vs. 1.218 cGy). The thyroid dose was 0 for ProBeam and 0.263 mGy for TrueBeam, as the thyroid was outside the KV‐CBCT irradiation range. In TomoTherapy MV imaging, Fine mode delivered twice the dose of Normal mode and three times that of Coarse mode. See Figure 4 for details.

### 3.2 Measurement of 5‐Year‐Old Child Phantom

In our study, we used a 5‐year‐old child phantom, similar to the adult phantom, focusing on four organs: the thyroid, lungs, breasts, and heart. Each organ was assessed at multiple points, with the absorbed dose for each organ tissue calculated as the mean of all measurement points for that specific organ.

**FIGURE 5:**
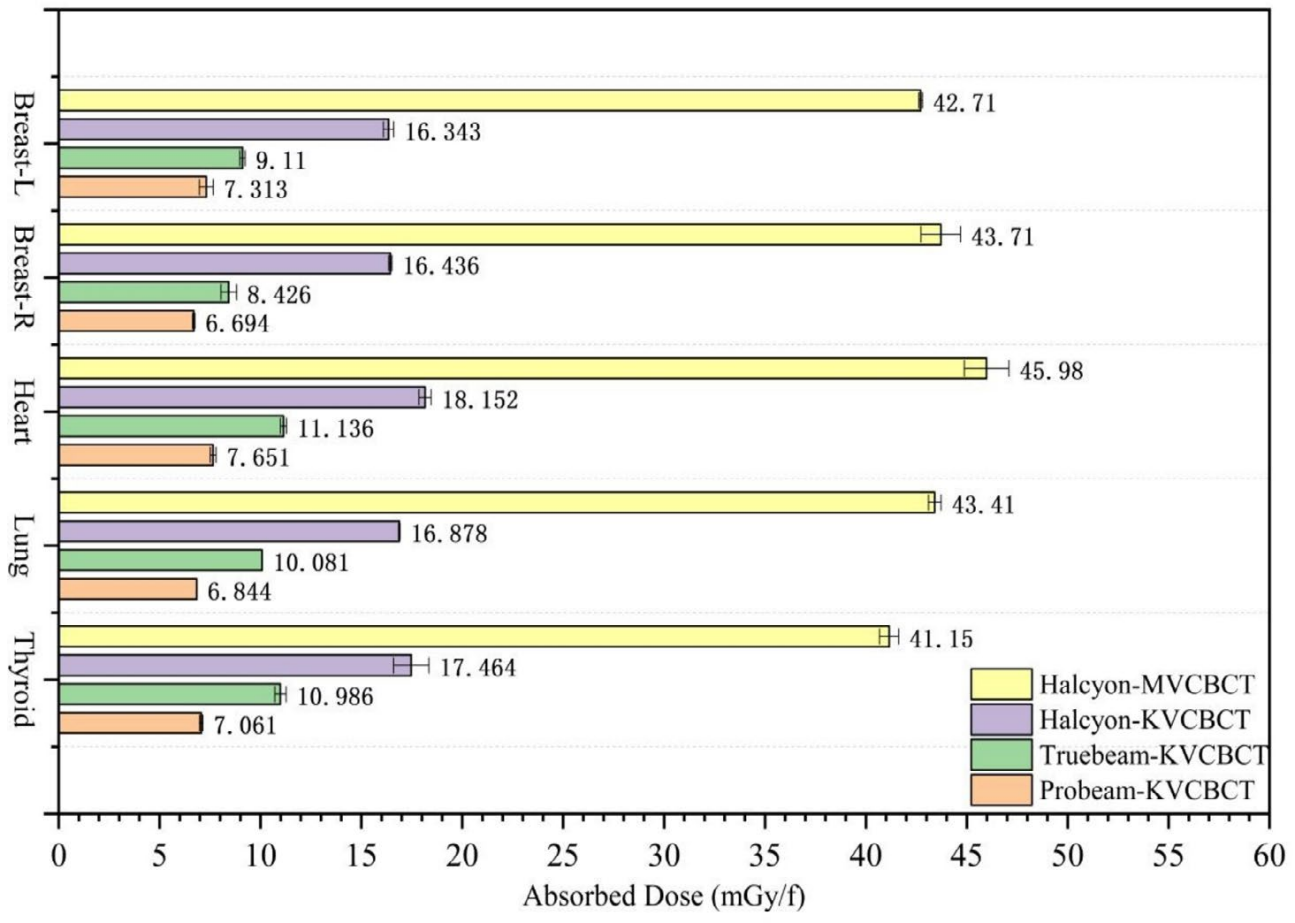
Absorbed dose in a 5‐year‐old child phantom induced by different imaging devices in IGRT.

The results from phantom measurements indicate that the absorbed dose induced by megavoltage‐imaging equipment is higher than that produced by kilovoltage‐imaging equipment. Among the kilovoltag‐ imaging devices, Halcyon yielded the highest absorbed dose in the phantom, with a single chest KV‐CBCT scan resulting in an absorbed dose of 16.878 mGy across both lungs. In the low‐dose mode of Halcyon, MV‐CBCT induced an absorbed dose of 4.341 cGy in both lungs of the phantom. See Figure 5 for details.

### 3.3 Image‐Guided Risk Assessment

The data obtained in this study were grouped according to different imaging devices and phantoms and accumulated based on the conventional treatment of 25 imaging doses, as shown in Table 2.

**TABLE 2:** Absorbed dose in the lungs of different phantoms from various imaging equipment.

Imaging method	5‐yo‐phantom (Gy,$\;\bar{x}$ ± SD)	Adult phantom (Gy,$\;\bar{x}$ ± SD)
Probeam‐KVCBCT	0.171 ± 0.001	0.102 ± 0.001
TrueBeam‐KVCBCT	0.252 ± 0.001	0.149 ± 0.002
Halcyon‐KVCBCT	0.422 ± 0.001	0.228 ± 0.004
Halcyon‐MVCBCT	1.085 ± 0.015	0.979 ± 0.005
TOMO‐coarse	—	0.245 ± 0.001
TOMO‐normal	—	0.469 ± 0.008
TOMO‐fine	—	0.927 ± 0.004

**FIGURE 6:**
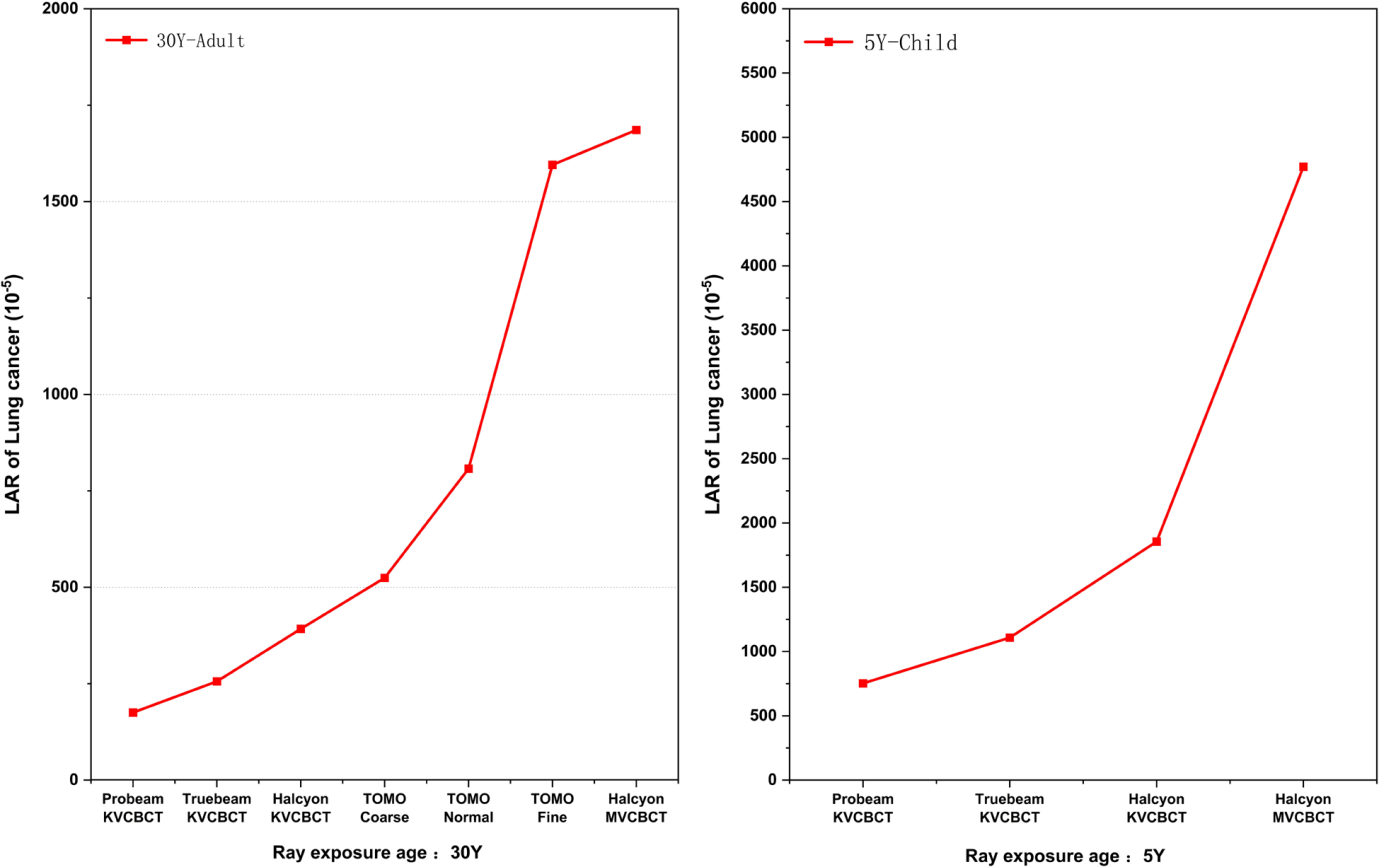
LAR of lung cancer caused by different imaging devices.

The LAR of lung cancer was evaluated based on the measured data, age at radiation exposure, and baseline lung cancer incidence rate in China, as shown in Figure 6.

## 4 Discussion

**FIGURE 7:**
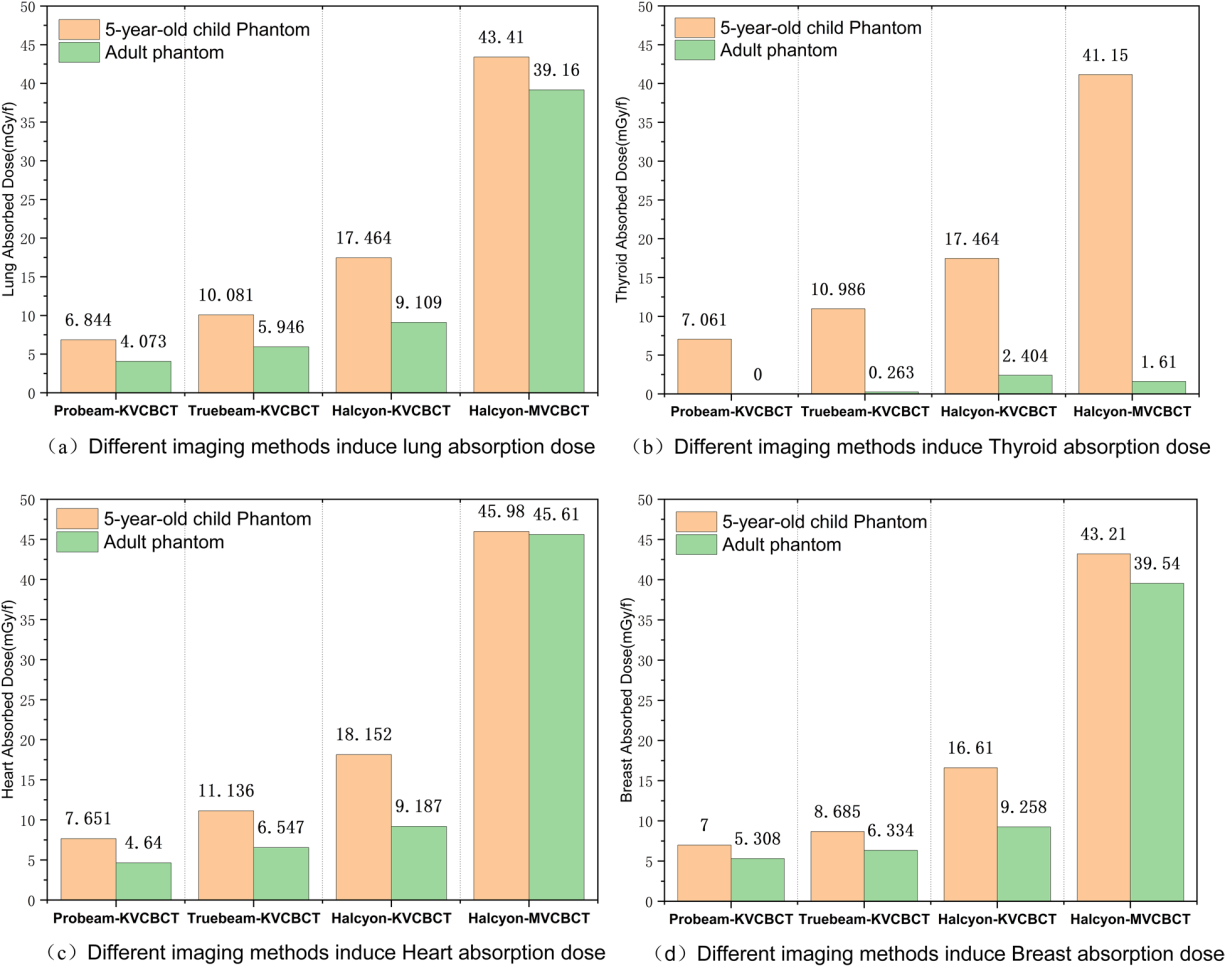
Absorbed doses in different organs of the 5‐year‐old child phantom and adult phantom induced by different imaging devices in IGRT and adult phantom induced by different imaging devices of IGRT.

This study employs OSLD as a measurement tool across various imaging devices, including TrueBeam, Halcyon, ProBeam, and TOMO. This contrasts with most other studies, which utilize TLD for the same purpose. The aim is to measure the absorbed dose in different organs of both adult and child phantom models under various chest imaging modes, as shown in Figure 7. In the adult model experiment, the dose from TrueBeam‐KVCBCT was about 1.5 times that of ProBeam‐KVCBCT, the dose from Halcyon‐KVCBCT was approximately 2.2 times higher than ProBeam‐KVCBCT, and the dose from Halcyon‐MVCBCT was significantly higher than the doses from the other three KVCBCT groups.

In the pediatric phantom experiments, the dose from TrueBeam‐KVCBCT was about 1.5 times that of ProBeam‐KVCBCT, and the dose from Halcyon‐KVCBCT was approximately 2.5 times that of ProBeam‐KVCBCT. Notably, the dose from Halcyon‐MVCBCT was significantly higher than that from the other three KVCBCT groups.

The imaging dose from a single CBCT scan exceeds 1 cGy, and at a distance of 25 cm from the central axis, the dose remains above 0.2 cGy. For a single IMRT session, the dose at 20 cm from the field edge is approximately 1 cGy, and at 25 cm, it is 0.4 cGy. The carcinogenic risk associated with diagnostic CT radiation has been widely acknowledged by both the academic community and the general public. For every additional 100 mGy of radiation, the risk of hematological malignancies doubles. A standard CT scan, with an average dose of approximately 8 mGy, can increase the risk of lymphatic or myeloid malignancies by about 16%.

In the field of radiotherapy, the scope of imaging guidance radiation is significant and encompasses multiple imaging sessions. Pediatric patients, under the same IGRT conditions, receive 2**–**3 times the imaging dose to key organs compared to adults. Given the potential for additional radiation exposure during radiotherapy, the risk of secondary tumors should not be underestimated, especially for younger pediatric patients, where potential risks should be carefully evaluated.

This study focused on KV‐CBCT and MV‐CBCT among various image guidance techniques, selecting only representative accelerators for investigation. Using a chest standard mode, scans were performed under the same simulation model to analyze their dosimetric differences. In practical applications, both image quality and guidance accuracy are of equal importance. Ideally, the lower the dose, the better, provided that the accuracy of image guidance is maintained. In future studies, when examining the dosimetric differences of various imaging devices, both image quality and guidance accuracy should be considered.

The 2007 AAPM‐TG75 report [16] provided recommendations for imaging dose management in IGRT. However, in clinical practice, only Halcyon integrates the dose from MVCBCT into patient treatment plans and performs dosimetric evaluation [17], whereas the vast majority of IGRT imaging doses are not included in the patient's dosimetric assessment and management. The U.S. Food and Drug Administration (FDA) issued industry guidance in June 2022 [18], requiring manufacturers to submit a performance evaluation report of their quantitative imaging technology before the radiological equipment is marketed.

## 5 Conclusions

In this study, we conducted a comprehensive measurement of absorbed doses within various organs of a phantom under image‐guided thoracic imaging modes using several prevalent medical electron linear accelerators and proton therapy systems. This was accomplished through optically stimulated luminescence dosimetry and a simulated human body model. The devices examined included the conventional “C‐arm” accelerator TrueBeam, the “annular” accelerator Halcyon, the “spiral tomotherapy” accelerator TOMO, and the advanced proton therapy system ProBeam.

Our findings indicate that daily thoracic CBCT can result in an absorbed dose of up to 1 Gy in thoracic organs. The radiation dose from CBCT in radiation therapy should not be overlooked, as a younger age at exposure is associated with an increased risk of secondary cancer development. Among the devices studied, ProBeam demonstrated the lowest dose and the lowest associated secondary cancer risk.

## Author Contributions

**Qinghao Cui:** investigation, methodology, writing – original draft preparation. **Xianrui Yan:** methodology, data curation. **Chengqiang Li:** methodology. **Jian Zhu:** funding acquisition, methodology, supervision. **Jun Ma:** methodology, supervision. **Tingting Liu:** funding acquisition, supervision, project administration.

## Conflicts of Interest

The authors declare no conflicts of interest.

## Data Availability

The data that support the findings of this study are not publicly available. Reasonable requests for access to the primary datasets and analysis code will be considered by the corresponding author.

## References

[1] S. Huang , Y. Xiao , H. Li , et al., “Research on Improving Radiotherapy Accuracy Based on Image‐Guided Radiotherapy,” Contrast Media & Molecular Imaging 2022 (2022): 9696403. 36034197 10.1155/2022/9696403 PMC9381236

[2] L. M. Force , I. Abdollahpour , S. M. Advani , et al., “The Global Burden of Childhood and Adolescent Cancer in 2017: An Analysis of the Global Burden of Disease Study 2017,” Lancet Oncology 20, no. 9 (2019): 1211–1225. 31371206 10.1016/S1470-2045(19)30339-0 PMC6722045

[3] A. J. Olch and P. Alaei , “How Low Can You Go? A CBCT Dose Reduction Study,” Journal of Applied Clinical Medical Physics 22, no. 2 (2021): 85–89. 33450139 10.1002/acm2.13164 PMC7882101

[4] E. Galamba , “Next‐Generation Therapies in Radiation Oncology,” Oncology Times 46, no. 4 (2024): 27.

[5] X. Sha , J. Duan , X. Lin , et al., “A New Proton Therapy Solution Provides Superior Cardiac Sparing Compared With Photon Therapy in Whole Lung Irradiation for Pediatric Tumor Patients,” Frontiers in Oncology 10 (2021): 611514. 33604292 10.3389/fonc.2020.611514 PMC7884855

[6] C. Bolan , “The Promise of Proton Therapy,” Journal of Applied Radiation Oncology 2, no. 1 (2013): 34–39.

[7] K. E and H. A , “Physical and Dosimetric Characteristic Properties of BeO OSL for Clinical Dosimetric Measurements,” Applied Radiation and Isotopes 186 (2022): 110199. 35544992 10.1016/j.apradiso.2022.110199

[8] S. Tsai , C. Chen , J. Lee , et al., “Evaluation of Effective Dose Using TLDs With Different Weighted PMMA Phantoms Undergoing Coronary Artery Calcium Computed Tomography Examination,” IEEE Transactions on Nuclear Science 60, no. 3 (2013): 2147–2154.

[9] C. Walter , J. Boda‐Heggemann , H. Wertz , et al., “Phantom and In‐Vivo Measurements of Dose Exposure by Image‐Guided Radiotherapy (IGRT): MV Portal Images vs. kV Portal Images vs. Cone‐Beam CT,” Radiotherapy and Oncology 85, no. 3 (2007): 418–423. 18023491 10.1016/j.radonc.2007.10.014

[10] J. C. A. Prestes , “Characterization of a 60Co Installation for Calibrating Clinics Dosimeters Used in Radiotherapy,” 2021.

[11] National Research Council, Division on Earth, Life Studies , et al., “Health Risks From Exposure to Low Levels of Ionizing Radiation: BEIR VII Phase 2,” 2006.

[12] M. Yanovskiy , Y. Y. Shaki , and Y. Socol , “Ethics of Adoption and Use of the Linear No‐Threshold Model,” Dose‐Response 17, no. 1 (2019): 500766316. 10.1177/1559325818822602 PMC6343444 30733652

[13] K. Ozasa , Y. Shimizu , A. Suyama , et al., “Studies of the Mortality of Atomic Bomb Survivors, Report 14, 1950–2003: An Overview of Cancer and Noncancer Diseases,” Radiation Research 177, no. 3 (2012): 229–243. 22171960 10.1667/rr2629.1

[14] H. Smith , “1990 Recommendations of the International Commission on Radiological Protection,” International Commission on Radiological Protection 21 (1991): 1–3. 2053748

[15] J. Ning , “Main Data of the Seventh National Population Census,” China Statistics 5 (2021): 4–5.

[16] M. J. Murphy , J. Balter , S. Balter , et al., “The Management of Imaging Dose During Image‐Guided Radiotherapy: Report of the AAPM Task Group 75,” Medical Physics 34, no. 10 (2007): 4041–4063. 17985650 10.1118/1.2775667

[17] Y. Huang , Y. Du , C. Li , et al., “Pediatric Cone Beam CT on Varian Halcyon and TrueBeam Radiotherapy Systems: Radiation Dose and Positioning Accuracy Evaluations,” Journal of Radiological Protection 39, no. 3 (2019): 739–748. 31042686 10.1088/1361-6498/ab1e74

[18] Administration UFAD , “Technical Performance Assessment of Quantitative Imaging in Radiological Device Premarket Submissions‐Guidance for Industry and FDA Staff,” 2022, FDA Guidance Document.

[19] Z. Yang , P. Zhao , and X. Wang “Compilation and Analysis of the Chinese Population Life Tables for 2000 and 2010: Based on Data From the Fifth and Sixth National Population Censuses,” Journal of Yunnan University of Finance and Economics (Social Science Edition) 27, no. 5 (2012): 67–69.

